# The reliability and accuracy of operational system data in a nationwide helicopter emergency medical services mission database

**DOI:** 10.1186/s12873-019-0265-y

**Published:** 2019-10-15

**Authors:** A. Heino, T. Iirola, L. Raatiniemi, J. Nurmi, A. Olkinuora, P. Laukkanen-Nevala, I. Virkkunen, M. Tommila

**Affiliations:** 1 FinnHEMS Research and Development Unit, Vantaa, Finland; 2 Department of Perioperative Services, Intensive Care Medicine and Pain Management, Turku University Hospital and University of Turku, Turku, Finland; 3 Emergency Medical Services, Turku University Hospital and University of Turku, Turku, Finland; 4 Centre for Pre-Hospital Emergency Medicine, Oulu University Hospital, Oulu, Finland; 5 Emergency Medicine Services, Helsinki University Hospital and Department of Emergency Medicine, University of Helsinki, Helsinki, Finland

**Keywords:** Clinical quality registry, HEMS, Pre-hospital, Documentation, Data reliability

## Abstract

**Aim:**

The aim of this study was to evaluate the reliability and accuracy of documentation in FinnHEMS database, which is a nationwide helicopter emergency service (HEMS) clinical quality registry.

**Methods:**

This is a nationwide study based on written fictional clinical scenarios. Study subjects were HEMS physicians and paramedics, who filled in the clinical quality registry based on the clinical scenarios. The inter-rater -reliability of the collected data was analyzed with percent agreement and free-marginal multi-rater kappa.

**Results:**

Dispatch coding had a percent agreement of 91% and free-marginal multi-rater kappa value of 0.83. Coding for transportation or mission cancellation resulted in an agreement of 84% and free-marginal kappa value of 0.68. An agreement of 82% and a kappa value of 0.73 for dispatcher coding was found. Mission end, arrival at hospital and HEMS unit dispatch -times had agreements from 80 to 85% and kappa values from 0.61 to 0.73. The emergency call to dispatch centre time had an agreement of 71% and kappa value of 0.56. The documentation of pain had an agreement of 73% on both the first and second measurements. All other vital parameters had less than 70% agreement and 0.40 kappa value in the first measurement. The documentation of secondary vital parameter measurements resulted in agreements from 72 to 91% and kappa values from 0.43 to 0.64.

**Conclusion:**

Data from HEMS operations can be gathered reliably in a national clinical quality registry. This study revealed some inaccuracies in data registration and data quality, which are important to detect to improve the overall reliability and validity of the HEMS clinical quality register.

## BACKROUND

Clinical quality registries have been established in many areas in health care to enable continuous quality management [1–3]. The data quality in these registries has to be assured, and plausible inaccuracies have to be identified to make the registries reliable as they are used in clinical quality management.

Physician-staffed units providing advanced pre-hospital critical care are part of emergency medical services (EMS) in many western countries. Collecting operational data from these services is considered an important part of quality control. Consensus-based guidelines on variables were published in 2011 [4]. Reliable documentation is necessary to achieve reliable data for scientific purposes and to standardise the operation protocols [5–7].

The FinnHEMS database, a Finnish clinical quality registry on Helicopter Emergency Medical Services (HEMS), was first introduced in 2011 and implemented nationwide in 2012. FinnHEMS database is the first nationally organised HEMS database in Europe. The database contains operational and clinical data of every HEMS mission in Finland. Previously, it has been shown that many data collection templates used in emergency care results in incoherent data, e.g. the Utstein template for reporting of cardiac arrest [8–10], and different templates for trauma data coding and scoring [11–17].

This study aimed to evaluate the reliability and accuracy of data documentation in a nationwide HEMS clinical quality registry, the FinnHEMS database. We hypothesised that there would be individual variation in registration habits in the FinnHEMS database. The results will help to improve the quality of data in clinical quality registries in pre-hospital critical care, as they will show the variables most prone to variation and imprecisions in registration, thus allowing to correct them by further instructions, training and data monitoring.

## Methods

### Setting

There are five physician-staffed HEMS units and one advanced paramedic-staffed HEMS unit in Finland. FinnHEMS is the national administrative unit in charge of organising the helicopter services and the development of HEMS operations. It is a non-profit organisation owned by all five Finnish university hospital districts. Operational and patient-related data from all HEMS missions nationwide has been gathered in this clinical quality registry since the beginning of 2012.

### Study design and participants

**Fig. 1:**
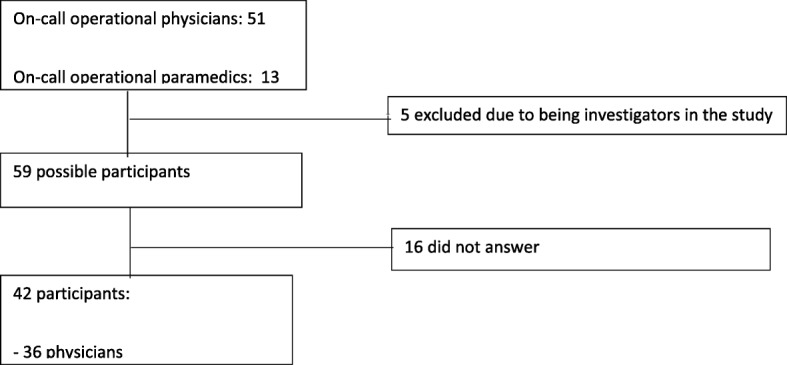
Study participants

This is a study of data collected from written fictional clinical scenarios. All FinnHEMS physicians (except the authors MT, TI, IV, JN and LR; *n* = 46) and paramedics (*n* = 13) working on-call in one of the six bases were invited to the study. The participants were anonymised to the investigators, but the home base of each participant was recorded. The participation rate was 71.2% (Fig. 1).

### Ethics

By Finnish legislation, no ethical approval was needed for this study because no patients were involved. Permission for the study was acquired separately from each university hospital. The clinical scenarios were fictional, and no actual patient data was used. Study subjects were informed of the study with two separate e-mails that were sent before the data collection began. Subjects filled the database on voluntary basis, and their approval to take part in this study was achieved as subjects filled the FinnHEMS database with their given personal identification number.

### Data collection

Six fictional HEMS missions within an imaginary 24-h HEMS duty were composed by principal investigators AH and MT, and they were approved by TI. The course of the duty, missions, patient characteristics and dispatch centre messages were described in a written story, attached with pre-hospital medical reports including EMS reports and HEMS reports (Additional file 1) imitating a real-life scenario in the Finnish EMS system [18, 19].

The scenarios included three HEMS missions with a single patient, one multi-patient mission with four patients and two missions where no patient was met or the mission was cancelled. The data registration based on the scenarios was piloted. A participant was asked to register the data based on the given documents. The study database was identical to the FinnHEMS database; the only difference was that the documentation was recorded on a different datafile.

Although representing very ordinary and potentially realistic HEMS missions, the scenarios (Additional file 1) were intentionally designed to be challenging and to reveal the possible weaknesses of the FinnHEMS database, based on the earlier FinnHEMS database user experiences and feedback. The prerequisite for the scenarios was full coverage of all sections in the FinnHEMS database while keeping the workload of the study participants reasonable.

E-mailed information, including data collecting period, study protocol and instructions to use of study database, was sent to all the participants three weeks before the data collection began, and the study documents were sent via post to all six bases. The material was sent to bases at the end of 2016 and they were asked to fill in the study database. The data collection period was from 1 December 2016 to 31 January 2017.

This study focused on operational data, including time variables and operational coding. All of this data was manually logged in the study database by study participants based on study material. This imitates real-life HEMS missions and FinnHEMS database. Operational data such as time variables are often used in pre-hospital studies and quality control, but the accuracy of these variables is rarely questioned. This study focused on the quality and accuracy of this data in the FinnHEMS database.

### Statistical analysis

To measure the inter-rater reliability, the per cent agreement and free-marginal multi-rater kappa were calculated. In per cent agreement, the number of equal variables among raters is divided with the number of overall variables that resulted, providing a measure of agreement between raters. Kappa is a form of correlation coefficient, and contrary to percent agreement, it considers a random agreement factor [20]. Free-marginal multi-rater kappa was used to study inter-rater reliability in this study setting for its suitability to studies that have free-marginal distributions, namely when raters do not know a priori the quantities of cases that should be distributed into each category [21–24]. Free-marginal multi-rater kappa can take values from 1 to − 1. Values from 0 to 1 indicate agreement better than chance, a value of 0 indicates a level of agreement that could have been expected by chance and values from − 1 to 0 indicate levels of agreement that are worse than chance.

## Results

Of the 59 invited HEMS participants, 42 were included in the final analysis (Fig. 1). Of these, 13 (31%) were female, and 29 (69%) were male. All six Finnish HEMS bases were represented, and study participant distribution among these was Vantaa with 10 participants (24%), Turku 9 (21%), Tampere 7 (17%), Oulu 4 (10%), Rovaniemi 6 (14%) and Kuopio 6 (14%).

### Mission coding

In this study, dispatch coding had least inter-rater variability (Table 1*).* Transportation or mission cancellation had most inter-rater variability based on free-marginal multi-rater kappa, and the use of cancellation codes X-0 (technical barrier) and X-9 (mission cancellation) especially seemed to vary. Dispatcher for HEMS unit can be one of the national dispatch centers, another EMS unit requesting support or the HEMS unit itself attending a mission. A per cent agreement of 82% and free-marginal multi-rater kappa value of 0.73 for dispatcher coding was achieved.

**Table 1:** Inter-rater reliability of Mission coding in FinnHEMS database

Coding^a^	Percent agreement, %	Free-marg.K [CI 95%]
Dispatch code	91	0.83 [0.52, 1.00]
Transportation/cancellation code	84	0.68 [0.36, 1.00]
Dispatcher	82	0.73 [0.23, 0.90]

^a^Code for HEMS unit attending a mission, registered in the FinnHEMS database by a participating physician or paramedic and based on the provided documents (Additional file 1)

### Time-related variables

At the mission’s end, the arrival at hospital and HEMS unit dispatch -times had per cent agreements from 80 to 85% and kappa values from 0.61 to 0.73 (Table 2). The emergency call to dispatch centre time had a per cent agreement of 71% and a kappa value of 0.56.

**Table 2:** Inter-rater reliability of Time-related variables in the FinnHEMS database

Time variable^a^	Percent agreement, %	Free-marg. K [CI 95%]
Emergency call	71	0.56 [0.37, 0.75]
HEMS unit dispatch	82	0.73 [0.46, 1.00]
HEMS unit en route	98	0.98 [0.95, 1.00]
On scene	99	0.99 [0.96, 1.00]
At patient	88	0.76 [0.53, 1.00]
Start of transportation	96	0.93 [0.88, 0.98]
Arrival at hospital	80	0.61 [0.26, 0.96]
Mission end	85	0.70 [0.44, 0.95]
HEMS unit free of mission	95	0.93 [0.87, 0.99]
Mission cancellation	89	0.83 [0.51, 1.00]

^a^The point of time in the mission, registered in the FinnHEMS database by a participating, attending physician or paramedic, based on the provided documents (Additional file 1)

### Vital parameters

The documentation of pain was the only parameter that had a percent agreement of 73% on both the first and second measurements. All other parameters had less than 70% percent agreement and a 0.40 kappa value in the first measurement (Table 3). According to the national HEMS CQR guidelines, the time point of the first measurement is the moment the patients has been met, and the secondary parameters are measured after treatment. The secondary vital parameter measurements resulted in per cent agreements from 72 to 91% and kappa values from 0.43 to 0.64.

**Table 3:** Inter-rater reliability of Vital parameters in the FinnHEMS database

Parameter^a^	Per cent agreement, %	Free-marg. K [CI 95%]
1. measurement^b^ 2. measurement^c^		
Cardiac rhythm	61	0.23 [− 0.11, 0.56]
	76	0.51 [0.17, 0.86]
Heart rate	60	0.19 [0.02, 0.37]
	82	0.64 [0.34, 0.94]
Blood pressure	69	0.38 [0.04, 0.73]
	77	0.54 [0.17, 0.91]
Respiration rate	65	0.30 [−0.11, 0.70]
	72	0.43 [0.01, 0.86]
Blood oxygen saturation (SpO_2_)	68	0.36 [0.03, 0.69]
	91	0.82 [0.72, 0.92]
Expiration carbon dioxide (etCO_2_)	62	0.24 [0.07, 0.42]
	76	0.52 [0.22, 0.82]
Pain	73	0.46 [0.31, 0.60]
	73	0.46 [0.17, 0.75]
GCS^d^	66	0.49 [0.16, 0.82]

^a^Vital parameter, registered in the FinnHEMS database by a participating physician or paramedic responsible for patient treatment at a HEMS mission and were based on the documents provided (Additional file 1)

^b^When patient was met

^c^After treatment

^d^GCS is documented only once

### Multi-patient mission documentation

Study scenarios included one mission with four patients: one severely injured patient was treated and transported by HEMS, and three other patients were triaged by the HEMS physician but transported by other EMS units. The severe patient was entered into the database by 41 of the 42 study participants whereas all four patients were registered by 23 of the 42 participants. During this study, there was no exact guidelines on multi-patient missions if all patients met by the HEMS unit should be registered in the CQR, or only those that were treated or transported by the HEMS unit.

### Documentation of adverse events

The FinnHEMS database documents adverse events in airway management. This study included one patient description which involved rapid sequence intubation. One study participant documented hypotension, eight participants documented hypotension with hypoxia as an adverse event on this mission, and 33 of the 42 participants documented that no adverse event followed the airway management.

## Discussion

This is the first study investigating the inter-rater agreement in an electronic nationwide clinical quality registry (CQR) in pre-hospital HEMS operations. The quality of operational documentation in this CQR is good and at some points even excellent. This finding promotes the use of CQR for internal system quality control and improvement as well as for scientific purposes. However, this study also reveals some deficits in the operational data of the HEMS CQR: these findings disclose the data most prone to variation and thus allows for improving the documentation.

First, transportation and cancellation coding had the lowest inter-rater agreement in mission coding, especially cancellation coding, which showed inter-rater unreliability. There are two cancellation codes used in the database; X-0 for mission denied due to technical reasons; and X-9; mission cancelled for patient-related reasons or after departure. The documentation of cancellation codes is based on the physicians or paramedics interpretation of given definitions and instructions, which increases the risk of individual variation. However, the codes also seem to be interpreted differently among HEMS bases, as the participants in some areas tend to register more X-0 codes at the expense of X-9. This discrepancy implies that local documentation habits can outweigh given instructions, which are nationally uniform.

Targeting HEMS units to complete properly selected missions is a key element in all HEMS operations, and for this reason, reliable documentation of cancelled missions and the reasons for cancellations is essential. Only by analysing accurate data on the underlying reasons is it possible to improve the accuracy of HEMS dispatch.

Time-related variables are often used for quality control and research; in some patient groups, such as patients with sudden cardiac arrest or major trauma, the incident to treatment delay (emergency call to a hospital) is one of the most important factors in measuring and improving the quality of EMS and HEMS. Nonetheless, among time-related variables, the time of emergency call had the lowest inter-rater reliability in this study setting, and also the documentation of HEMS unit dispatch, mission end and arrival at hospital times showed only moderate inter-rater reliability.

The registration of time points may seem simple, but again the documentation is based on the interpretation of national guidelines for the usage of CQR, which may vary, and no definitions of variables were not included in the e-mails or material sent to study participants. Indeed, it is likely that varying personal conceptions of the definitions of specific time points are the primary reason for inaccurate documentation. For example, in cases where another EMS unit asks the HEMS unit to join a mission, it might be unclear whether to register the time of emergency as the call time of the original call to the dispatch centre or the time when the other EMS unit calls. In addition to exact instructions, proper guidance and continuous quality control of documentation are very important.

Surprisingly, major deficits were found in vital parameter documentation. Overall inter-rater reliability was poor for vital parameters. The study setting and registration of these parameters based on written documents can expound on part of this unreliability, but it can also be hypothesised that the correct point in the mission to register the first and second vital measurements remains unclear. This could explain especially the poor reliability found for second measurements when compared to first measurements. Instruction to perform a measurement when the patient is met, is an exact time point, whereas there can be major differences in whether the second vital parameter of a patient is measured at the beginning of treatment, at the end of treatment or just before transportation of a patient. This uncertainty is a marked fault in the documentation and should be addressed when improving HEMS QCR as vital parameters are essential parts of quality control, post assessment of patient management and data for clinical research.

Documenting possible adverse events is an important part of high-quality health care. For example, recording complications in pre-hospital airway management is encouraged, and a template already exists. Our study focused on overall documentation in HEMS CQR, and adverse events had a small quota in this study; thus, no comprehensive conclusions on their reliability can be made. The same limitation applies to documentation of multi-patient missions.

Our study revealed inaccuracies in the documentation that can be raised with adjustment to instructions, staff education and the continuous monitoring of data. The accuracy of vital parameter and time-related variable documentation could benefit from data gathered automatically by monitoring devices, although these devices have their limitations especially for their usage in unconventional pre-hospital setting. With these adjustments, the accuracy and quality of the marked operational data in HEMS CQR can be further improved to an even higher level to better serve pre-hospital studies and the development of HEMS system.

## Limitations

This study was based on written fictional mission scenarios, which can never equal a real-life pre-hospital setting where an actual patient is seen and treated. The written description of the scenarios may give space for individual understandings, and registrations based only on written material in several documents may lead to more inaccuracies related to interpretation of the materials than the inherent accuracy of registration. Especially on the multi-patient mission description, when no precise guideline was found during data collection period for multi-patient mission registrations. As the initial hypothesis was that there are individual differences in the use of the clinical quality register, the missions and patient descriptions may have been written in a way that leads to differences and inaccuracies. However, it can be presumed that this study setting still disclosed most of the defects in the national HEMS CQR, and there are not necessarily as many inaccuracies in real-life operational data.

## Conclusion

Based on this study, data from HEMS operations can be gathered reliably in a national CQR. This study, by using written patient scenarios, revealed some inaccuracies in data registration and data quality, which are important for detecting how to improve the overall reliability and validity of the HEMS CQR. Routine, intrinsic evaluations of CQRs are important and recommended for quality control in all healthcare registries.

## Supplementary information

**Additional file 1.** Written fictional mission descriptions, EMS reports and HEMS reports.

## Data Availability

See Additional file 1.

## Abbreviations

CQR: Clinical quality registry
EMS: Emergency medical service
HEMS: Helicopter emergency medical service
