# Supplementary material for: The reliability and accuracy of operational system data in a nationwide helicopter emergency medical services mission database
Source: BMC Emerg Med. 2019 Oct 15;19:53. doi: 10.1186/s12873-019-0265-y (PMC6792230; doi:10.1186/s12873-019-0265-y)
Supplement: Supplementary file 1 — Additional file 1. Written fictional mission descriptions, EMS reports and HEMS reports. [file 12873_2019_265_MOESM1_ESM.pdf]

ADDITIONAL FILE 1. Written fictional mission descriptions, EMS reports and HEMS reports.

Study period: 24-hour shift on Saturday 17-9-2016 to Sunday 18-9-2016

For missions 2., 3., 5. and 6. please see the attached FH- and SV210-forms.

Not all variables have been filled in on the FH and SV 210 forms to enable us to observe how respondents apply information gathered from different sources. However, the FinnHEMS database is to be completed as comprehensively as possible according to the information given and at respondent's discretion.

Duty begins at 9:00 at the base

At 9:30 gear check and maintenance activities begin

At 10:12 Study town's emergency response centre gives the call-out for mission 1.

Mission 1.

Study town's emergency response centre dispatches on 17.9.2016 at 10.13:

"A700 Care home Iltatähti, Tutkimuskuja 3 [Study Alley 3], Study town; call began at 10:12; care home nurses performing resuscitation; ETU01, ETU3221, FH90"

Description of the situation:

FH90 departs on the mission with copter at 10:18. At 10:22 FH90 is still on the way to the scene and ETU01 calls for FH90 on Virve radio (Used by Finnish Fire and Rescue, Police and Emergency Medical Service): "Here's a 91-year-old male patient with numerous primary diseases and advanced Alzheimer's. DNR decision exists. Last seen in bed awake half an hour ago. Primary rhythm asystole."

FH90 instructs to stop resuscitation and cancels the mission. ETU01 and 3221 handle the rest of the situation. FH90 turns back to the station at 10.24.

At 10:33 back at the base with copter, gear check and maintenance of gear continues

At 11:30 gear check and maintenance completed

At 11:35 registration of mission 1 begins

At 11:41 registration of mission 1 completed.

At 14:10 ETU1221 calls for FH90 on Virve and requests backup: "At *the railway station of study town mission B202, there's a patient with no pulse, ventricular fibrillation, resuscitation initiated, will you join the mission?"*

At 14:10 FH 90 calls emergency response center and asks for FH90 to join ETU 1221's mission.

At 14:11 Study town's emergency response centre dispatches on mission 2.

Mission 2. + ATTACHMENTS (FH-form and SV 210)

Study town's emergency response centre dispatches on 17.9.2016 at 14:11:

"B202 At the railway station, Tutkimuskatu [Study street], Study town, beginning of call 14:00; a small Fiat has crashed into a lamp post, driver in the car; ETU1221, RTU4125, FH90"

Description of the situation:

FH 90 departs on the mission with copter at 14:15. FH90 is at the scene at 14:27 and encounters the patient at 14:29.

A small private car has crashed into a lamp-post. The front bumper a little crushed, no other damage to the car. Driver's door is open, the patient lying down next to the car. The paramedic of ETU1221 is ventilating with breathing bag, I-Gel inserted. No external signs of trauma.

Advance information from the paramedic of ETU 1221: "A 72-year-old male, Pasi Ryhänen according to driving license. Found sitting in the car with seatbelt on. Safety devices didn't go off. Wasn't breathing, no pulse. Was removed from the vehicle and resuscitation commenced. The first analysis of rhythm at 14:08, then VF and shocked. Sinus rhythm restored at second shock and ROSC 12 min after beginning of call. IO access established but no drugs administered."

After ROSC haemodynamics unstable. Instructed by the FH 90 physician: the ETU 1221 paramedic inserts an i.v. cannula at the bend of the elbow and noradrenaline-infusion is initiated with it. Patient's level of consciousness is still low, keeps his eyes closed, makes no sounds, flexes on pain symmetrically with limbs on both sides. Makes spontaneous efforts at respiration. Decision to intubate with sedation, in addition an arterial cannula is inserted.

The patient is transported by ambulance to Y9 (university hospital of study town) escorted by the physician. Patient enrolled and handed over to the ED at 15:30. In this context information on patient's primary diseases: "Hypertension, prostate cancer operated on in 2008 and in remission."

At 16:00 back at base, supplies replenished

At 16:15 registration of mission 2 begins

At 16:18 Study town's emergency response center dispatches on mission 3.

Mission 3. + ATTACHMENTS (FH form and SV 210s)

Study town's emergency response centre dispatches on 17.9.2016 at 16:18:

"A203 freeway ramp, study town; RTA two cars, head-on collision, four affected, 80 km/h speed limit; ETU1241, ETU1231, ETU2221, RTU331, RTU231, FH90"

Description of the situation:

FH 90 departs on the mission with copter at 16:24. ETU1231 the first EMS unit at the scene and on the orders of the EMS supervisor, the paramedic of ETU1231 is the on-scene medical commander. FH90 at the scene at 16:32.

At the scene in its own lane front facing forward a green Audi, front bonnet crushed, airbags activated, driver in the car, rescue detaching, back doors open. In addition, in front of the Audi a red Skoda in the ditch on its left side, bonnet to bonnet with Audi, front bonnet crushed, driver's side of the car crushed, safety devices activated, driver in the car, rescue detaching.

L5 gives further information: " One red patient (Rami Eskola, 27 years) in the Skoda, one yellow in the Audi (Mirja Juusela, 38 years) and two green in the Audi (Kyllikki Juusela 69 years and Erkki Juusela 74 years). The green ones got out of the car by themselves and called the emergency response centre. The red patient is breathing, thin radial pulse, unalert. The yellow one awake, radial pulse present, no pain, can't get out of the car by herself. ETU2221 treats the green woman. ETU1241 treats the green man. We will treat the yellow driver once released. Will you treat the red patient?"

FH90 doc quickly goes round the other patients, instructs on pain medication and transport with trauma pre-alert to the university hospital. The doc takes a photo with a cell phone of the other patients' SV210 forms. After this the doc moves to the red patient who is just being transferred by rescue onto a spine board, a collar is in position. In prone position the patient opens his eyes to speech, makes sounds and indicates pain sites. Is very pale and hypotensive, cold peripherally. Intra-abdominal bleeding suspected. According to "Load and go" principle taken to Y9 by helicopter. By road from the accident scene to Y9 110 km, estimated transportation time by copter 20 min.

Before transportation the FH90 doc establishes i.v. access on the patient. During transportation eFAST and administration of pain medication by FH90 doctor. Pre-alert on the patient to Y9 ED.

At 17:05 FH90 is on hospital helipad and at 17:15 the patient has been handed over to the ED at study town university hospital.

At 17:45 back at base by copter

At 18:00 replenishing of supplies

At 18:15 registration of mission 2. continues

At 18:30 registration of mission 2. completed, and registration of mission 3 begins

At 18:59 registration of mission 3. completed

At 19:37 study town's emergency response centre dispatches on mission 4

Mission 4.

Study town's emergency response centre dispatches on 17.9.2016 at 19:37

"A700 Tutkimuskuja 2 [Study alley 2], Study town; beginning of call 19:33; not breathing; ETU3221, ETU4882, FH90"

Description of the situation:

At 19:39 the copter is being moved on the dolly from the hangar, the copter's engine not running. The emergency response centre informs: "Advising units en route for Tutkimuskuja, mission code changed to C772"

At 19:40 FH90 doc states that (s)he isn't going on the mission after the change of code and begins the registration of mission 4.

At 19:50 registration of mission 4. completed.

Day changes Sun 18.9.2016

At 00:18 ETU3221 consults FH 90 physician about giving medication, they are on a B772-mission. The patient is a 2.5 year-old boy with developmental delays and epilepsy. The parents have given 5 mg of midazolam buccally and the paramedics have given 2.5 mg x 3 i.v. of Stesolid. Regardless of this, the seizure continues, has already lasted for 15 min. In light of phone talk FH90 doc decides to join the mission at 00:21.

At 00:22 FH90 physician calls study town's emergency response centre and reports that the code for ETU3221's mission has changed to A772. Requests that FH90 join the mission.

At 00:23 Study town's emergency response centre dispatches on mission 5.

Mission 5. + ATTACHMENTS (FH-form and SV210)

Study town's emergency response centre dispatches on 18.9.2016 at 00:23

"A772 Tutkimuskatu 10 [Study street 10], Study town; beginning of call 00:06; 2-year-old child patient, epilepsy; ETU 3221, FH90"

Description of the situation:

FH 90 departs on the mission with ground unit at 00:23. The ground unit is used because weather prevents flying the copter. The patient is encountered on the way along the freeway at 00:42. Patient's seizure has abated, lying on his side eyes closed, breathing spontaneously without problems. Doesn't open eyes, makes sounds, reacts to pain with movement of both pairs of limbs. Gaze strays to the right. Stesolid 2.5 mg i.v. also administered.

Transportation continues at 00:44, FH 90 physician escorts with ETU 3221. The patient is lying on his side, breathing spontaneously. At 00:45 physician calls the paediatrician on-call at the study town university hospital, who advises to go directly to paediatric ICU. When approaching the hospital, the patient is clearly waking up, opens eyes and seems to be making eye contact, makes sounds and moves all limbs. According to the mother the situation already seems very normal, in any case the child can't yet speak. The patient is handed over to the paediatric ICU at 01:30 in good condition.

At 01:38 returning with ground unit from mission 5, when study town's emergency response centre dispatches on mission 6

Mission 6. + ATTACHMENTS (FH-form)

Study town's emergency response centre dispatches on 18.9.2016 at 01:50.

"A752, Hämäräkuja 16 F 78 [Dark street 16 F 78], study town; injected with fentanyl, not breathing; ETU1221, ETU01, FH90"

Description of the situation:

FH90 responds to the mission while on return journey at 01:50. The scene is close by so the journey continues by car. Arrival at the scene as the first EMS unit at 01:54, patient encountered at 01:55. The patient is a youngish male "Pate", identity not established. A friend says that "Pate" injected fentanyl shortly before the beginning of emergency call. When "Pate" lost consciousness and his face began to turn blue the friend had called the emergency response centre.

Patient's respiration rate is 4/min. FH90 doc initiates mask ventilation. ETU1221 and ETU01 are soon assisting at the scene. I.V. access is established, and he gets 0.4 mg i.v. naloxone on doc's orders. With this, the patient quickly regains consciousness and starts breathing spontaneously. Vital signs are ok, no external trauma discovered. The patient totally refuses to go anywhere for treatment. Says that he accidentally injected too much fenta and promises not to inject anything more. 0.8 mg i.m. of naloxone is administered and the scene becomes X-6 [patient refuses transportation] at 02:17.

At 02:42 back at base with ground unit. Supplies replenished.

At 02:59 replenishing of supplies completed.

At 03:12 registration of mission 5. is started

At 03:25 registration of mission 5. completed and the registration of mission 6. is begun

At 03:50 registration of mission 6. completed

At 09:00 report to the next doc on call

At 09:30 the shift ends

MISSION 2.

(1/2)

FinnHEMS **HEMS report**

☐ Kuopio ☐ Tampere	☐ Oulu ☐ Turku	☐ Rovaniemi ☐ Vantaa ☒ STUDY BASE	Mission ID	Dispatch code ??	Transp / canc code ??
Date 17.9.2016	Scene address TUTKIMUSKATU	☐ = home address	Emerg call 14:00	Amb ETU 1221	☐ Ground unit ☒ Other
Patient name RYHÄNEN PASI		Municipality TUTKIMUSKAUPUNKI	Alarm		??
Identity number 72 YEARS OLD	Home municipality / country TUTKIMUSKAUPUNKI	Dispatcher ??	On scene		14:27
Hospital Y 9	Reported/ scene ☒ Police ☐ Family Tel	Point of departure (Station) other	At patient		14:29
			Transp start		14:57
Transportation ☒ Amb + ☒ DOC/param. ☐ Helicopter ☐ Other		HBS 0-8 ??	At hospital		15:20
			Free		15:30
Physician/ paramedic STUDY PHYSICIAN			Mission end		??

Description of the situation

PATIENT COLLIDED IN HIS CAR WITH A LAMP-POST. LOW TRAUMA ENERGY. NO PULSE WHEN MET BY ETU 1221, PRIMARY RHYTM VF.

Primary impression

ETU 1221 ALS RESUSCITATION, I-GEL PLACED, VENTILATES WELL. 2X DEFIBRILLATED. ROSC 12 MINUTES. RR 120/60, FA 140, SpO2 99, NO SPONTANEOUS BREATHING. SYMMETRICAL, SMALL PUPILS, NO REACTION TO LIGHT. ECG SHOWS FA 102.

Response to treatment

ANAESTHETIC INTUBATION (VIDEOLARYNGOSCOPE + BOUGIE), IO AND IV ACCESS, NORADRENALINE INFUSION, ARTERIAL LINE + PRESSURE MONITOR.

Primary diseases, home medication

NOT KNOWN.

Trauma patient

☒ open fracture

☐ fracture

X fracture / laceration / tenderness

O deep/ penetrating injury

Ø no findings

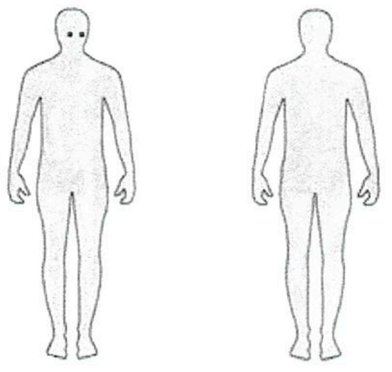

Working diagnosis RESUSCITATION WITH ROSC

GCS	14:29				
Eye-opening 1-4<br>4 spontaneously<br>3 to speech<br>2 to pain<br>1 no response	1				
Verbal response 1-5<br>5 oriented<br>4 confused<br>3 inappropriate words<br>2 sounds<br>1 no response	1				
Motor response 1 - 6<br>6 obeys commands<br>5 moves to pain<br>4 withdrawal from pain<br>3 abnormal flexion<br>2 abnormal extension<br>1 no response	1				
Total	3				

Resuscitation

☐ Witnessed OHCA ?

☐ During treatment

☐ Unwitnessed OHCA

☐ Bystander CPR

☐ No ACLS, reason

☐ Temporary ROSC at

Primary rhythm

☒ VF ☐ PEA

☐ ASY ☐ Other

Delays since the start of call (min):

First response FinnHEMS

1.DC 8 ROSC 12

, permanent at 14:12

Acute coronary event

Ecg at

Ecg at

Pain started app. at

Continuous pain at

Delay of thrombolysis min thrombolysis at

since the start / aggravation of pain ☐ contraindication check

☐ PCI/ UAP -treatment ☐ Cardiolog consultation

MISSION 2.

Pain o ~80 kg

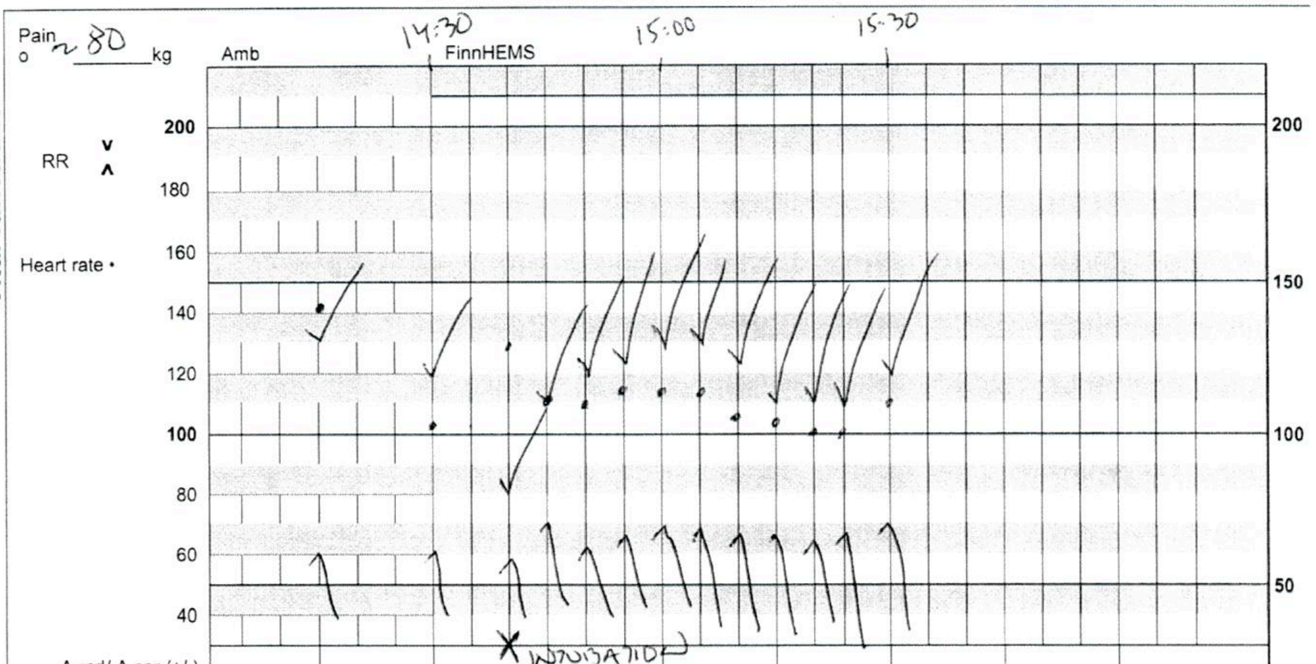

Vital functions

A.rad/ A.car (+/-)					
Respiration rate	12	14	16	16	16
Breath sounds		+/+			
Rhythm	FA		FA		FA
$EtCO_2$ (kPa)	4,9	5,8	5,6		4,2
SpO_2 (%)	99	84 99	97	97	97
Gluc / pain				6,2 / –	
Temperature					

Yhteensä

Medication

alfentan./ fentan. (mg)	0,2 ; 0,1
morphine/ oxycodone (mg)	
adrenaline (mg)	
propofol (mg)	50
suxamethonium (mg)	
rocuronium (mg)	80
EFEDRIN (MG)	9
noradr. 0,04 mg/ml (ml/h)	10 ml / hour
propofol 10/20 mg/ml (ml/h)	10 ml / hour

Fluids

Ringer/ ~~NaCl~~ 500 ml	
Ringer/ ~~NaCl~~ 500 ml	
Kolloidi ml	

Interventions

Blood gases time 14 : 45 pH 7,19 pCO_2 6,0 pO_2 12,0 BE 5.0 Hb 141 K 3.8 Na 139

☒ Art.can.rad.dx/ sin ☐ NGT, no_ ☒ io-access: tibia/ humerus dx/ sin ☐ Other STUDY PHYSICIAN

Intubation/ SAD nro 8.0, attempts 1, depth 24 cm, time 14:40 Intubator. Resp.mode: CPAP/ BiPAP/ IPPV/ SIMV FiO_2 50 % V. vol: 500 ml Fr: 14 /min IPAP___ EPAP/PEEP 5

Kela

Service provider and VAT number: MISSION 2.

Report and claim for reimbursement for patient transportation

OSA 1

Identity number	Travel date	Unit	Running no
72 YEARS OLD	17.09.16	ETU 1221	

Subscriber: [X] dispatch center [ ] other, what?
Origin: [ ] station [ ] other, what?

Scene address: [ ] = home address [ ] Onward transp
TUTKIMUSKATU 1, TUTKIMUSKAUPUNKI

Dispatch code: B 202

Transp / canc code: 222

Driving km total:

Emergency call: 1400

Patient name: RYHÄNEN PASI

Mission start: 1401

On scene: 1406

Home address (street and city):

Patient on additional place [ ] on stretchers [ ] sitting

Home municipality: [ ] Foreigner / form attached

At patient: 1407

Reference no:

Transport: 1457

Patient released: 1530

Mission end:

Cause of transport:
- [X] Illness or pregnancy
- [X] Motor vehicle accident *) Registration number ABC 123
- [ ] Accident at work *)

*) Insurance company

No Kela reimbursement: [ ] Care facility / hospital patient [ ] Other

To be invoiced where?

	Euros
Basic fee -20 km	
Invoiceable extra kilometres km	
2. ambulance driver h min	
Waiting time (over 1 hr) h min	
In total	
Excess	
Invoiced from Kela	

Description of the situation. Primary reason (symptom or seizure/arrest, mechanism of injury; time of start or event), eye witness's contact information)

A PASSER-BY TELEPHONED. A SMALL FIAT HAD COLLIDED WITH A LAMP-POST. THE DRIVER IS IN THE VEHICLE. SPEED LIMIT AT THE SITE 40 KM/HOUR. ACCORDING TO AN EYEWITNESS THE SPEED OF THE VEHICLE WAS LESS THAN THIS.

First-response unit on scene

Primary impression (symptom, trauma-related findings)

THE PATIENT SEATED IN THE DRIVER'S SEAT IN SEATBELT. AIRBAGS NOT ACTIVATED. SMALL DENT IN THE FRONT BUMPER BAR. NO OTHER DAMAGE TO CAR. PATIENT PULSELESS WHEN MET.

Diseases, current medication, drug allergies, previous hospital treatment: NOT KNOW.

MONITORING AT	BLOOD PRESS.	HEART RATE	RHYTHM	RESPIRAT. RATE	RESPIRAT. AUSCULT.	PEF	$ETCO_2$	SpO_2	CONSCIOUSNESS (GCS) Eyes	Verbal	Motor	PAIN 0-10	B-gluc	ALC. METER	TEMPERATURE. from where
When met A 14:08	—		VF												
B 14:12	130/62	140	FA				6,9	99	1	1	1				
C 14:30	120/60	102	FA				6,6	99	1	1	1				
Patient handover D 15:30	120/70	110	FA				4,2	97	1	1	1		6,2		T.OTO 35,2 °C

Treatment (interventions, medication) and **response.** [ ] Consultation with physic. [X] Physician on scene. Physician's name and position. Instructions for treatment: FH90 STUDY PHYSICIAN

REMOVED FROM THE VEHICLE ONTO THE ROAD, CPR INITIATED. AT 14:08 VF, 1× DEFIBRILLATION → CPR 2 MINUTES. I-GEL SIZE 4 AT 14:09, VENTILATES WELL. AT 14:10 VF, 1× DEFIBR. CPR 2 MINUTES. AT 14:11 IO-ACCESS TO RIGHT HUMERUS, RINGER 500 ML IO. AT 14:12 ROSC, FA. GCS STILL DEPRESSED, NO SPONTANEOUS BREATHING. ANAESTHETIC INTUBATION BY FH 90 PHYSICIAN.

Signature of person refusing treatment / transportation

[ ] Information continues on another sheet

Signature and number of person giving treatment | Numbers of other personnel | Name and phone number of next of kin | [ ] Accompanied

DECLARATION OF THE HEALTH CARE FACILITY

I hereby declare based on the previous report that the patient's physical condition [ ] requires [ ] doesn't require ambulance transportation.

Signature, clarification of signature and position of the person receiving the patient

Name and stamp of the receiving facility: Y 9

SV 210 04.07

MISSION 3

(1/2)

FinnHEMS **HEMS report**

			Mission ID	Dispatch code	Transp / canc code
☐ Kuopio ☐ Tampere	☐ Oulu ☐ Turku	☐ Rovaniemi ☐ Vantaa ☒ STUDY BASE		A203	?

Date	Scene address	☐ = home address	Emerg call 16:17	Amb	☐ Ground unit ☒ Other
17.9.2016	VALTAVÄYLÄ				

Patient name	Municipality			
ESKOLA RAMI	TUTKIMUSKAUPUNKI	Alarm		16:18

Identity number	Home municipality / country	Dispatcher			
27 YEARS OLD	TUTKIMUSKAUPUNKI	HÄKE	On scene		16:32

Hospital: Y9

Reported/ scene: ☒ Police ☐ Family

Tel

Point of departure: (Station) / other

At patient		16:35
Transp start		16:45
At hospital		17:05
Free		17:15
Mission end		??

Transportation: ☐ Amb + ☐ DOC/param. ☒ Helicopter ☐ Other

HBS 0-8: ??

Physician/ paramedic: STUDY PHYSICIAN

Description of the situation

HEAD-ON COLLISION INVOLVING 2 CARS. ONE THE RIGHT WAY UP IN THE DITCH, THE OTHER ON ITS LEFT SIDE IN THE DITCH. 4 PERSONS AFFECTED.

Primary impression

PATIENTS TRIAGET: 2 x GREEN, 1x YELLOW, 1X RED. RED PATIENT THE DRIVER OF SKODA, ALONE IN THE VEHICLE, SEATBELT ON. CAR ON ITS LEFT SIDE, EXTENSIVELY OUT OF SHAPE. RESCUE RELEASES PATIENT, UNCONSCIOUS IN CAR, OPENS EYES AS SET TO SPINE BOARD, MOVES HANDS, GROANS.

Response to treatment

"LOAD AND GO" BY HELICOPTER, I.V. ACCESS -> REHYDRATION REGAINS CONSCIOUSNESS. e FAST EN ROUTE & BLOOD AROUND THE LIVER.

Primary diseases, home medication

NUT ALLERGY, OTHERWISE HEALTHY.

Trauma patient

☒ open fracture

☐ fracture

X fracture / laceration / tenderness

O deep/ penetrating injury

Ø no findings

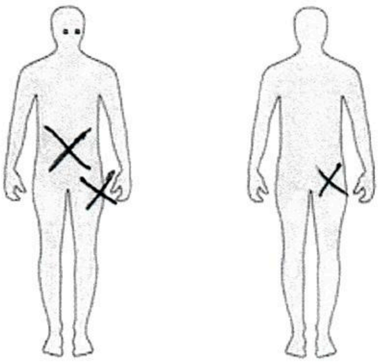

Working diagnosis: BLOOD IN THE ABDOMEN

GCS	16:37	16:42	17:00	
Eye-opening 1-4<br>4 spontaneously<br>3 to speech<br>2 to pain<br>1 no response	4	4	4	
Verbal response 1-5<br>5 oriented<br>4 confused<br>3 inappropriate words<br>2 sounds<br>1 no response	2	4	5	
Motor response 1 - 6<br>6 obeys commands<br>5 moves to pain<br>4 withdrawal from pain<br>3 abnormal flexion<br>2 abnormal extension<br>1 no response	5	6	6	
Total	11	14	15	

Resuscitation

☐ Witnessed OHCA

☐ During treatment

☐ Unwitnessed OHCA

☐ Bystander CPR

☐ No ACLS, reason

☐ Temporary ROSC at

Primary rhythm

☐ VF ☐ PEA

☐ ASY ☐ Other

Delays since the start of call (min):

First response FinnHEMS

1.DC ROSC

, permanent at

Acute coronary event

Ecg at

Ecg at

Pain started app. at

Continuous pain at

Delay of thrombolysis min thrombolysis at

since the start / aggravation of pain ☐ contraindication check

☐ PCI/ UAP -treatment ☐ Cardiolog consultation

MISSION 3.

Vital functions

Pain o ~85 kg | Amb | 16:30 FinnHEMS | 17:00 | 17:30

RR v ^

Heart rate •

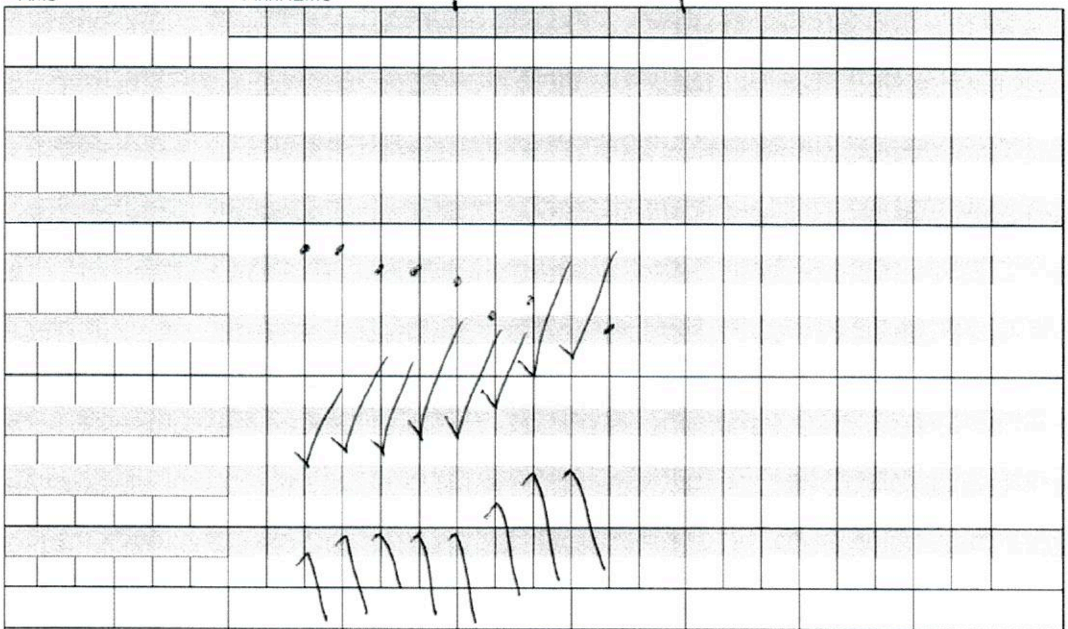

	Amb	FinnHEMS						Yhteensä
A.rad/ A.car (+/-)								
Respiration rate		28						
Breath sounds		+/+						
Rhythm			SINUS, TACHYCARDIA					
$EtCO_2$ (kPa)								
SpO_2 (%)			92		94	96		
Gluc/ pain			9		6	4		
Temperature								

Medication

								Yhteensä
alfentan./ fentan. (mg)								
morphine/ oxycodone (mg)			5					
adrenaline (mg)								
propofol (mg)								
suxamethonium (mg)								
rocuronium (mg)								
noradr.______ mg/ml (ml/h)								
propofol 10/20 mg/ml (ml/h)								

Fluids

Ringer/~~NaCl~~ 500 ml		↑————						
Ringer/~~NaCl~~ 500 ml								
Kolloidi ______ ml		|————						

Interventions

Blood gases time____:____pH__________pCO2__________pO2__________BE__________Hb__________K__________ Na__________

☒ Art.can.rad.dx/ sin ATTEMPT, DOESN'T SUCCEED ☐ NGT, no__ ☐ io-access: tibia/ humerus dx/ sin ☒ Other eFAST 16:49 = BLOOD IN ABDOMEN

Intubation/ SAD nro__________, attempts__________, depth__________cm, time__________ Intubator, Resp.mode: CPAP/ BiPAP/ IPPV/ SIMV FiO_2__________% V. vol:__________ ml Fr:__________/min IPAP____ EPAP/PEEP________

Kela

Service provider and VAT number: MISSION 3

Report and claim for reimbursement for patient transportation

OSA 1

**Identity number**	**Travel date**	Unit	Running no
38 YEARS OLD	1 7 0 9 1 6	ETU 1231/ L5	

Subscriber [X] dispatch center [ ] other, what?

Origin [ ] station [ ] other, what?

Scene address [ ] = home address [ ] Onward transp: VALTAVÄYLÄ, OHITUSTIEN RAMPPI, TUTKIMUSKAUPUNKI

Dispatch code: A 203

Transp / canc code:

Driving km total

Emergency call: 1 6 1 8

Patient name: JUUSELA MIRJA

Mission start: 1 6 1 8

On scene: 1 6 2 4

Home address (street and city):

Patient on additional place [ ] on stretchers [ ] sitting

Home municipality: TUTKIMUSKAUPUNKI

[ ] Foreigner / form attached

At patient: 1 6 2 4

Reference no:

Transport:

Cause of transport

[ ] Illness or pregnancy

[X] Motor vehicle accident *) Registration number AUDI XXX-123

[ ] Accident at work *)

*) Insurance company: OTHER PARTICIPANT SKODA AAA-123

Patient released:

Mission end:

No Kela reimbursement

[ ] Care facility / hospital patient [ ] Other

To be invoiced where?

	Euros
Basic fee -20 km	
Invoiceable extra kilometres km	
2. ambulance driver h min	
Waiting time (over 1 hr) h min	
**In total**	
Excess	
Invoiced from Kela	

Description of the situation. Primary reason (symptom or seizure/arrest, mechanism of injury; time of start or event), eye witness's contact information)

HEAD-ON COLLISION INVOLVIN 2 CARS. 4 PERSONS AFFECTED. AUDI ON THE ROAD RIGHT WAY UP. SKODA IS LEFT SIDE IN THE DITCH. BOTH BONNETS BADLY OUT OF SHAPE. 100 KM/HOUR IN THE AREA

First-response unit on scene:

Primary impression (symptom, trauma-related findings)

TWO PATIENTS TRIAGED GREEN ON THE BACK SEAT EXITED THE CAR UNAIDED BY THEMSELVES. AMBULATORY, NO PAIN. AUDI DRIVER TRIAGED YELLOW STUCK ON THE FRONTSEAT, CONSCIOUS, NO PAIN. SKODA DRIVER TRIAGED RED, STUCK, NO RESPONSE, BREATHING, A. RAD+.

Diseases, current medication, drug allergies, previous hospital treatment

MONITORING AT	BLOOD PRESS.	HEART RATE	RHYTHM	RESPIRAT. RATE	RESPIRAT. AUSCULT.	PEF	$ETCO_2$	SpO_2	CONSCIOUSNESS (GCS) Eyes	Verbal	Motor	PAIN 0-10	B-gluc	ALC. METER	TEMPERATURE, from where
When met A 16:25	A.RAD+	~100		~20					4	5	6	0			
B															
C															
Patient handover. D															

Treatment (interventions, medication) and **response.** [ ] Consultation with physic. [X] Physician on scene. Physician's name and position. Instructions for treatment

FH90 STUDY PHYSICIAN

FH 90 → RED PATIENT

ETU 1231 → YELLOW PATIENT (MIRJA)

ETU 2221 → GREEN PATIENT (JUUSELA KYLLIKKI)

ETU 1241 → GREEN PATIENT (JUUSELA ERKKI)

Signature of person refusing treatment / transportation

[ ] Information continues on another sheet

Signature and number of person giving treatment | Numbers of other personnel | Name and phone number of next of kin | [ ] Accompanied

DECLARATION OF THE HEALTH CARE FACILITY

I hereby declare based on the previous report that the patient's physical condition [ ] requires [ ] doesn't require ambulance transportation.

Signature, clarification of signature and position of the person receiving the patient

Name and stamp of the receiving facility

49

SV 210 04.07

Kela

Service provider and VAT number: MISSION 3.

Report and claim for reimbursement for patient transportation

OSA 1

Identity number	Travel date	Unit	Running no
74 YEARS OLD	170916	ETU 1241	

Subscriber ☐ dispatch center ☐ other, what? Origin ☐ station ☐ other, what? Scene address ☐ = home address ☐ Onward transp

Dispatch code: A 203

Transp / canc code

Emergency call: 1618

Patient name: JUUSELA ERKKI

Mission start: 1619

Driving km total

Patient on additional place ☐ on stretchers ☐ sitting

On scene: 1627

Home address (street and city)

Home municipality ☐ Foreigner / form attached

At patient: 1628

Reference no

Transport

Cause of transport
☐ Illness or pregnancy
☒ Motor vehicle accident *) Registration number XXX-123 AUDI
☐ Accident at work *)
*) Insurance company

Patient released

Mission end

No Kela reimbursement
☐ Care facility / hospital patient ☐ Other
To be invoiced where?

	Euros
Basic fee -20 km	
Invoiceable extra kilometres km	
2. ambulance driver h min	
Waiting time (over 1 hr) h min	
In total	
Excess	
Invoiced from Kela	

Description of the situation. Primary reason (symptom or seizure/arrest, mechanism of injury; time of start or event), eye witness's contact information)

HEAD-ON COLLISION INVOLVING 2 CARS.
THE PERSON ON THE REAR SEAT OF THE UPRIGHT CAR, EXITED UNAIDED. TRIAGED AS GREEN.

First-response unit on scene

Primary impression (symptom, trauma-related findings)

WALKING. PAIN-FREE. LUCID.

Diseases, current medication, drug allergies, previous hospital treatment

MONITORING AT	BLOOD PRESS.	HEART RATE	RHYTHM	RESPIRAT. RATE	RESPIRAT. AUSCULT.	PEF	$ETCO_2$	SpO_2	CONSCIOUSNESS (GCS) Eyes	Verbal	Motor	PAIN 0-10	B-gluc	ALC. METER	TEMPERATURE, from where
When met A 16:28	A. RAD+								4	5	6	0			
B															
C															
Patient handover. D															

Treatment (interventions, medication) and **response.** ☐ Consultation with physic. ☒ Physician on scene. Physician's name and position. Instructions for treatment

FH 90 STUDY PHYSICIAN

Signature of person refusing treatment / transportation

☐ Information continues on another sheet

Signature and number of person giving treatment | Numbers of other personnel | Name and phone number of next of kin | ☐ Accom-panied

DECLARATION OF THE HEALTH CARE FACILITY

I hereby declare based on the previous report that the patient's physical condition ☐ requires ☐ doesn't require ambulance transportation.

Signature, clarification of signature and position of the person receiving the patient

Name and stamp of the receiving facility

SV 210 04.07

Kela

Service provider and VAT number

MISSION 3.

Report and claim for reimbursement for patient transportation

OSA 1

Identity number	Travel date	Unit	Running no

Subscriber | dispatch center | other, what? | Origin | station | other, what? | Scene address | = home address | Onward transp | Dispatch code

Emergency call 1618	Patient name JUUSELA KYLLIKKI		Transp / canc code
Mission start 1619		Patient on additional place on stretchers sitting	Driving km total
On scene 1626	Home address (street and city)	Home municipality	Foreigner / form attached
At patient 1626		Reference no	

Transport	Cause of transport<br>☐ Illness or pregnancy<br>☒ Motor vehicle accident *) Registration number XXX-123<br>☐ Accident at work *)<br>*) Insurance company	No Kela reimbursement<br>☐ Care facility / hospital patient ☐ Other<br>To be invoiced where?	Basic fee -20 km	Euros
Patient released			Invoiceable extra kilometres km	
Mission end			2. ambulance driver h min	
			Waiting time (over 1 hr) h min	

Description of the situation. Primary reason (symptom or seizure/arrest, mechanism of injury; time of start or event), eye witness's contact information)

ON THE BACK SEAT AT THE TIME OF THE CRASH, COLLISION INVOLVING 2 CARS.

In total	
Excess	
Invoiced from Kela	

First-response unit on scene

Primary impression (symptom, trauma-related findings)

WALKING. NO PAIN.

Diseases, current medication, drug allergies, previous hospital treatment

MONITORING AT	BLOOD PRESS.	HEART RATE	RHYTHM	RESPIRAT. RATE	RESPIRAT. AUSCULT.	PEF	$ETCO_2$	SpO_2	CONSCIOUSNESS (GCS) Eyes	Verbal	Motor	PAIN 0-10	B-gluc	ALC. METER	TEMPERATURE, from where
When met A 16:26	A. RAD +							100	4	5	6	0			
B															
C															
Patient handover. D															

Treatment (interventions, medication) and **response.** ☐ Consultation with physic. ☒ Physician on scene. Physician's name and position. Instructions for treatment

FH 90 STUDY PHYSICIAN

Signature of person refusing treatment / transportation

☐ Information continues on another sheet

Signature and number of person giving treatment	Numbers of other personnel	Name and phone number of next of kin	☐ Accom-panied

DECLARATION OF THE HEALTH CARE FACILITY	**I hereby declare** based on the previous report that the patient's physical condition ☐ requires ☐ doesn't require ambulance transportation.<br>Signature, clarification of signature and position of the person receiving the patient	Name and stamp of the receiving facility<br>Y 9

SV 210 04.07

MISSION 5.

FinnHEMS HEMS report

☐ Kuopio ☐ Tampere ☐ Oulu ☐ Turku ☐ Rovaniemi ☐ Vantaa ☒ STUDY BASE	Mission ID	Dispatch code ??	Transp / canc code ??
Date 18.9.2016 Scene address TUTKIMUSKATU 10 ☒ = home address	Emerg call 00:06	Amb	☒ Ground unit ☐ Other
Patient name HIMANEN JAAKKO Municipality TUTKIMUSKAUPUNKI	Alarm	00:07	??
Identity number 2 YEARS OLD Home municipality / country TUTKIMUSKAUPUNKI Dispatcher ?	On scene		
Hospital Y9 Reported/ scene ☐ Police ☒ Family Tel Point of departure Station/ other	At patient		00:42
	Transp start		00:44
Transportation ☒ Amb + ☒ DOC/param. ☐ Helicopter ☐ Other HBS 0-8 ??	At hospital		01:25
	Free		01:30
Physician/ paramedic STUDY PHYSICIAN	Mission end		??

Description of the situation

EPILEPTIC. SEIZURE PERSISTED IN SPITE OF MEDICATION.

Primary impression

ETU 3221 ENCOUNTERED ON FREEWAY, PATIENT BREATHING SPONTANEOUSLY, KEEPING EYES CLOSED, MAKING NO SOUNDS, GAZE DEVIATES TO RIGHT, FLEXES UPPER LIMBS TO PAIN. 2.5 MG IN DIAZEPAM (STESOLID) IS ADMINISTERED → IMPROVEMENT IN GAZE

Response to treatment

TRANSPORTATION TO Y9 LYING ON SIDE. PEDIATRIC ICU CONSULTATED → TRANSPORTATION DIRECTLY PICU. PATIENT IMPROVING, WAKING UP, MAKING EYECONTACT

Primary diseases, home medication

NEUROLOGICAL (MENTAL) DELAY + EPILEPSY. MEDICATION RECENTLY CHANGED. MOTHER CAN'T REMEMBER NAME OF IT.

Trauma patient

☒ open fracture
☐ fracture
X fracture / laceration / tenderness
O deep/ penetrating injury
Ø no findings

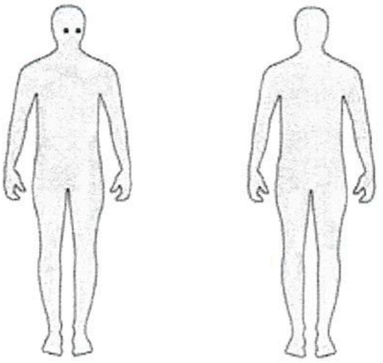

Working diagnosis EPILEPSY

GCS					
Eye-opening 1-4<br>4 spontaneously<br>3 to speech<br>2 to pain<br>1 no response	?				
Verbal response 1-5<br>5 oriented<br>4 confused<br>3 inappropriate words<br>2 sounds<br>~~1 no response~~					
Motor response 1 - 6<br>6 obeys commands<br>5 moves to pain<br>4 withdrawal from pain<br>3 abnormal flexion<br>2 abnormal extension<br>1 no response		[illegible]			
Total					

Resuscitation

☐ Witnessed OHCA
☐ During treatment
☐ Unwitnessed OHCA
☐ Bystander CPR
☐ No ACLS, reason
☐ Temporary ROSC at

Primary rhythm

☐ VF ☐ PEA
☐ ASY ☐ Other

Delays since the start of call (min):

First response FinnHEMS
1.DC ROSC
, permanent at

Acute coronary event

Ecg at
Ecg at
Pain started app. at
Continuous pain at
Delay of thrombolysis min thrombolysis at
since the start / aggravation of pain ☐ contraindication check
☐ PCI/ UAP -treatment ☐ Cardiolog consultation

[MISSION 5.]

Vital functions

Pain
o ________kg

Amb | FinnHEMS

RR v ^

Heart rate •

200 | 180 | 160 | 140 | 120 | 100 | 80 | 60 | 40

200 | 150 | 100 | 50

A.rad/ A.car (+/-)								
Respiration rate								
Breath sounds								
Rhythm								
$EtCO_2$ (kPa)								
SpO_2 (%)								
Gluc / pain								
Temperature								
								Yhteensä

Medication

alfentan./ fentan. (mg)								
morphine/ oxycodone (mg)								
adrenaline (mg)								
propofol (mg)								
suxamethonium (mg)								
rocuronium (mg)								
noradr.______ mg/ml (ml/h)								
propofol 10/20 mg/ml (ml/h)								

Fluids

Ringer/ NaCl _______ ml								
Ringer/ NaCl _______ ml								
Kolloidi _______ ml								

Interventions

Blood gases time_____:_____pH__________pCO_2__________pO_2__________BE__________Hb__________K__________ Na________

☐ Art.can.rad.dx/ sin ☐ NGT, no__ ☐ io-access: tibia/ humerus dx/ sin ☐ Other

Intubation/ SAD nro_______________, attempts__________, depth________________cm, time____________ Intubator_ Resp.mode: CPAP/ BiPAP/ IPPV/ SIMV FiO_2_________% V. vol:__________ ml Fr:__________/min IPAP____ EPAP/PEEP ________

Kela | Service provider and VAT number: MISSION 5.

Report and claim for reimbursement for patient transportation — OSA 1

Identity number	Travel date	Unit	Running no
2 YEARS OLD	18091 6	ETU 3221	

Subscriber ☐ dispatch center ☐ other, what? | Origin ☐ station ☐ other, what? | Scene address ☐ = home address ☐ Onward transp | Dispatch code

Emergency call 0006	Patient name HIMANEN JAAKKO	Scene address: TUTKIMUSKATU 10 TUTKIMUSKAUPUNKI	Transp / canc code
**Mission start** 0007		Patient on additional place ☐ on stretchers ☐ sitting	**Driving km total**
On scene 0012	Home address (street and city)	Home municipality ☐ Foreigner / form attached: TUTKIMUSKAUPUNKI	
At patient 0013		Reference no	
Transport	Cause of transport: ☐ Illness or pregnancy	No Kela reimbursement ☐ Care facility / hospital patient ☐ Other	Basic fee -20 km / Euros
Patient released	☐ Motor vehicle accident *) Registration number	To be invoiced where?	Invoiceable extra kilometres km
	☐ Accident at work *)		2. ambulance driver h min
**Mission end**	*) Insurance company		Waiting time (over 1 hr) h min

Description of the situation. Primary reason (symptom or seizure/arrest, mechanism of injury; time of start or event), eye witness's contact information)

EPILEPTIC, MOTHER WAKENED BY NOISE, FOUND HAVING A SEIZURE IN BED. MOTHER GAVE 5 MG OF BUCCAL MIDAZOLAM, THEN MADE THE EMERGENCY CALL.

In total | Excess | Invoiced from Kela

First-response unit on scene

Primary impression (symptom, trauma-related findings)

LYING ON SIDE IN BED, BREATHING, LIMBS STILL TWITCHING. IV ACCESS AT 00:15, DIAZEPAM 2.5 MG AT 00:16 AND ADDITIONAL DOSE 2.5 MG AT 00:18; CALL TO FH 90 PHYSICIAN AND ONCE MORE DIAZEP. 2.5 MG 00:20 -> SEIZING STILL PERSISTS

Diseases, current medication, drug allergies, previous hospital treatment

NEUROLOGICAL (MENTAL) DELAY + EPILEPSY. MEDICATION CHANGED 2 WEEKS AGO. LAST SEIZURE 6 MONTHS AGO.

MONITORING AT	BLOOD PRESS.	HEART RATE	RHYTHM	RESPIRAT. RATE	RESPIRAT. AUSCULT.	PEF	ETCO2	SpO2	CONSCIOUSNESS (GCS) Eyes	Verbal	Motor	PAIN 0-10	B-gluc	ALC. METER	TEMPERATURE, from where
When met A															
B															
C															
Patient handover. D															

Treatment (interventions, medication) and **response.** ☐ Consultation with physici ☒ Physician on scene. Physician's name and position. Instructions for treatment

AT 00:22 SEIZURES CEASED, PATIENT SLEEPY, BREATHING SPONTANEOUSLY, FH 90 PHYSICIAN ON SCENE AT 00:42. PATIENT'S GAZE DEVIATES TO THE RIGHT -> 2.5 MG OF DIAZEPAM IV. AT 01:20 PATIENT IMPROVES, HANDOVER TO HOSPITAL UNPROBLEMATIC.

Signature of person refusing treatment / transportation

☐ Information continues on another sheet

Signature and number of person giving treatment	Numbers of other personnel	Name and phone number of next of kin	
		HIMANEN SARI	☒ Accompanied

DECLARATION OF THE HEALTH CARE FACILITY

I **hereby declare** based on the previous report that the patient's physical condition ☐ requires ☐ doesn't require ambulance transportation.

Signature, clarification of signature and position of the person receiving the patient

Name and stamp of the receiving facility: Y 9 PEDIATRIC ICU

SV 210 04.07

FinnHEMS **HEMS report**

MISSION 6.

☐ Kuopio ☐ Tampere	☐ Oulu ☐ Turku ☒ STUDY	☐ Rovaniemi ☐ Vantaa BASE	Mission ID	Dispatch code A752	Transp / canc code X-6
Date 18.9.2016	Scene address HÄMÄRÄKUJA 16 F 78	☐ = home address	Emerg call 01:48	Amb ETU 1221	☒ Ground unit ☐ Other
Patient name "PATE"		Municipality TUTKIMUSKAUPUNKI	Alarm		01:50
Identity number APROX. 20 YEAR OLD (NOT KNOWN)	Home municipality / country	Dispatcher HAKE	On scene		01:54
Hospital X-6	Reported/ scene ☐ Police ☐ Family Tel	Point of departure Station (other)	At patient		01:55
			Transp start		
Transportation ☐ Amb + ☐ DOC/param. ☐ Helicopter ☐ Other		HBS 0-8 ??	At hospital		
			Free		02:17
Physician/ paramedic STUDY PHYSICIAN			Mission end		??

Description of the situation

A FRIEND REPORTS THAT "PATE" HAD INJECTED FENTANYL. THERE HAD BEEN OTHERS PRESENT, LEFT THE SCENE. "PATE'S" IDENTITY NOT EXACTLY KNOWN.

Primary impression

SLUMPED UNCONSCIOUS IN AN ARMCHAIR, FACE BLUEISH, BREATHING SPARSE -> MASK VENTILATION INITIATED & SpO_2 40 -> SpO_2 90, HR 40 -> 88, BP 100/50, GLUC 6.3, IV ACCESS -> NALOXONE 0,4 MG IV -> PATIENT WAKES UP

Response to treatment

AFTER NALOXONE WAKES UP, IS LUCID. BP 122/79, HR 92, SpO_2 97. TOTALLY REFUSES ANY TREATMENT. NEUROLOGY OK. NO INJURIES. ADDITIONAL 0,8 MG NALOXONE IV, STAYS WITH FRIEND.

Primary diseases, home medication

HEPATITIS C

Trauma patient

☒ open fracture
☐ fracture
X fracture / laceration / tenderness
O deep/ penetrating injury
Ø no findings

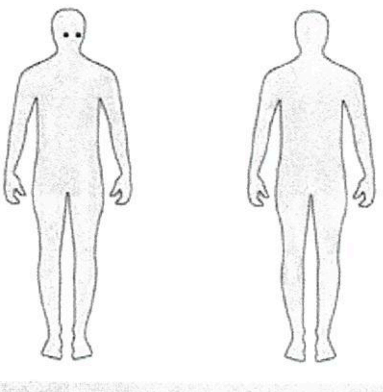

Working diagnosis INTOXICATION

GCS	01:55	02:10			
Eye-opening 1-4: 4 spontaneously, 3 to speech, 2 to pain, 1 no response	1	4			
Verbal response 1-5: 5 oriented, 4 confused, 3 inappropriate words, 2 sounds, 1 no response	1	5			
Motor response 1 - 6: 6 obeys commands, 5 moves to pain, 4 withdrawal from pain, 3 abnormal flexion, 2 abnormal extension, 1 no response	1	6			
Total	3	15			

Resuscitation

☐ Witnessed OHCA
☐ During treatment
☐ Unwitnessed OHCA
☐ Bystander CPR
☐ No ACLS, reason
☐ Temporary ROSC at

Primary rhythm
☐ VF ☐ PEA
☐ ASY ☐ Other

Delays since the start of call (min):
First response FinnHEMS
1.DC ROSC
, permanent at

Acute coronary event

Ecg at
Ecg at
Pain started app. at
Continuous pain at
Delay of thrombolysis min thrombolysis at
since the start / aggravation of pain ☐ contraindication check
☐ PCI/ UAP -treatment ☐ Cardiolog consultation
